# Alleged Cure of Rheumatism

**Published:** 1846-06

**Authors:** 


*Alleged Cure for Rheumatism.*—A worthy, kind-hearted gentleman assured us the other day, that he was in possession of a never failing remedy for rheumatism, in all its protean forms. It was this: carry a bit of brimstone in the pantaloons pocket, habitually, and never be without it. On asking for an explanation of the principle upon which the disease was either cured or kept at bay, he at once solved the problem by saying the sulphur was absorbed by the blood, the seat of the malady!—*Boston Med. and Surg. Jour.*

